# Human Cord Blood ILCs – Unusual Like My Career as a Scientist

**DOI:** 10.3389/fimmu.2021.752283

**Published:** 2021-10-28

**Authors:** Sabrina Bianca Bennstein

**Affiliations:** Institute for Transplantation Diagnostics and Cell Therapeutics, Medical Faculty, Heinrich-Heine University Düsseldorf, Düsseldorf, Germany

**Keywords:** NK cell, innate lymphoid cells (ILCs), cord blood (CB), women, development

## 1 Introduction

When I was in junior middle school, we were asked to make a collage about how we imagined our lives would look like at the age of 30. Given that for a teenager turning 30 was - at least for me - a great life milestone, I pictured myself in full bloom of an adult, potentially at the peak of my career. Hence, my collage had a woman in the center and everything else, such as activities, friends, career, and romantic relationships, were in a circle around the woman (the future me!). I guess I put myself in the middle, as I was raised to believe that I could accomplish anything if I tried hard enough and am determined to give it my best.

However, when I grew older, I realized that this general view I had, was not shared by everyone. Even though I successfully completed my PhD just recently, the journey towards it was not a smooth ride. I encountered several difficulties, especially as some people perceived and discouraged me based on my unusual *curriculum vitae* (CV) but also based on my gender. Given my strong belief to follow what I love to do and my unwillingness to accept these opinions, I faced the odds and challenges. However, I feel if this happened to me, this might also happen to others, who might be discouraged to follow their dreams.

Hence, when I read the call of this special issue, I immediately felt I would like to contribute to this topic and take the opportunity to share my story – the achievements and in particular the challenges – to make a statement, but also to encourage others in similar situations to follow their own path.

## 2 Achievements

After completing my A-levels, I started an apprenticeship as biological technical assistant. I was fascinated by natural science since visiting the Bernhard-Nocht Institute for Tropical Medicine in Hamburg during a school trip. However, I could not picture what life as a scientist would be like and if I enjoy working in a lab. Therefore, I did not study immediately, but trained as technician. I worked as a technician for four years, became fascinated by immunology, and my work was honored by several co-authorships (1–6). However, at the age of 25, I decided I would like to do more than conducting experiments for others. I wanted to understand the results, get a bigger picture, and most importantly, write the publications myself.

Hence, I started my undergraduate studies in Molecular Biomedicine at the Rheinische Friedrich-Wilhelms-Universität Bonn in 2013 at the age of 25, which was four to seven years older than the average student. To further strengthen my immunological background, I studied Integrated Immunology at the University of Oxford in 2016 at the age of 28. Here, I published my first review focusing on the development of murine invariant natural killer T cells (7).

As my interest in unusual immune cell subsets grew, I started my PhD in 2017 at the age of 29 focusing on human innate lymphoid cells (ILCs) in Germany.

### 2.1 Human Innate Lymphoid Cells in Umbilical Cord Blood

Starting my PhD in a scientifically young field, which was also new territory for the group, was an additional challenge. Hence, one dives into literature and at first, the ILC field seemed very straightforward: Human ILCs have a lymphoid morphology and no rearranged receptors on the cell surface (8), they are classified into three different subsets, which mirror T cell functionality (9): ILC1 secreting IFNγ and expressing TBET (10), ILC2 secreting IL-4, IL-5, and IL-13 (11), and ILC3 secreting IL-22 and IL-17A (12) depending on their NKp44 expression (13). When studying the role of human ILCs closely, dual – beneficial as well as harmful – roles have been described during different pathological settings, whereas especially tissue-resident IL-22 secreting human NKp44^+^ILC3 seemed to be highly beneficial in gastrointestinal transplantations, preventing GvHD after HSCT, and might decrease morbidity in patients with IBD [reviewed by us (14)]. However, later it became obvious that human ILCs are not so straightforward after all.

We started to look at ILCs in umbilical cord blood (CB), as human ILC developmental pathways and their direct precursors are not yet fully understood, and hardly any data existed on circulating ILCs at that time. Even though some publications showed CB ILC data (15–19), no study focused entirely on the phenotype, transcriptome, or functional capacity of human CB ILCs, although CB ILC frequencies were significantly higher compared to circulating ILCs from children and adults, they are still rare and usually have a frequency of less than 1% (20). Of note, we could later show total ILC frequencies drop even further in elderly adults (21) potentially due to a migration into tissues during inflammatory processes by cytokines or chemokines, as suggested (21, 22).

#### 2.1.1 Cord Blood ILCs Are Not Like Their Tissue-Resident Counterparts

In order to study CB ILCs in more detail, we developed a flow cytometry staining panel for their faithful identification (23). Given that ILCs are very rare and ILC1s were, and still are, the least well-defined subset, the composition of the lineage (lin) cocktail is essential to exclude unwanted cell populations, especially in precursor enriched sources such as CB. In the next step we focused on the functional capacity of CB ILCs. Surprisingly, unlike tissue-resident ILCs, we observed CB ILC1 and ILC3 to be mostly unresponsive to the classical interleukin stimuli reported in literature, except for a small subpopulation of CD161^+^ILC1 (21), while CB ILC2 were fully functional (24). It took the first 6-month of my PhD to accept as well as believe in these results and to rule out any possible experimental issues. As we were new to the field, we could not believe this at first. Once convinced, we renamed CB ILC1 and ILC3 to ILC1-like and ILC3-like cells to stress the fact that they are not identical compared to their tissue counterparts (21).

#### 2.1.2 Cord Blood ILC3-like Cells May Play an Important Role for Pre-Birth Innate Defense

Nevertheless, in line with tissue-resident ILC3 (25), CB ILC3-like cells were able to secrete LIF when additional IL-2 was added. LIF has been described to be vital for implantation and maintenance of pregnancies and trophoblast differentiation suggesting an important role of CB ILC3-like cells for pregnancies homeostasis (26, 27). Strikingly, CB ILC3-like cells were fully functional when stimulated by an experimental TLR2:1 ligand Pam_3_CSK_4_ and IL-2 (24). The capacity to sense conserved pathogen-associated molecular patterns (PAMPs) is a typical innate feature and suggests that CB ILC3-like cells might be vital for the first line of defense against different pathogens (28) passing the placental barrier, such as *Toxoplasma gondii* (29), which is a threat to the fetus health. This is also of basic interest, as CB ILC3-like cells as well as PB ILC3, also known as ILC precursor (ILCP), have been described to differentiate into all three ILC subsets and NK cells (15) suggesting some precursors might have multiple potential of innate immune defenses, homeostasis as well as differentiation capacity.

#### 2.1.3 Cord Blood ILCs Have a Unique Transcriptional Profile

We further conducted the first bulk transcriptomic analyses of CB ILCs. The three CB ILC subsets shared nine differentially expressed genes compared to CD56^bright^ NK cells. One of these genes, the transcription factor (TF) inhibitor of DNA binding 3 (ID3) caught our attention, as the helix-loop-helix TF ID2 has been previously assumed to constitute a major TF for murine and human ILCs (18, 30, 31). Upon further analysis, we observed a unique *ID3/ID2* ratio > 1 within CB ILCs (24), but not in CB NK cells, PB ILCs taken from a published data set (32), or tonsillar ILC3. This unusual *ID3/ID2* ratio > 1 was also observed in CB CD4^+^ T cells, but not in CB CD8^+^ T cells or both PB T cell subsets (24) suggesting that CB ILCs may have different, unique transcriptional profiles compared to PB ILCs and might have a shared developmental pathway with T cells, potentially within the thymus. In line with this, CB ILCs, especially CB ILC1-like cells, clustered closer to T cells, while CB ILC3-like cells clustered closer to NK cells (24).

#### 2.1.4 Cord Blood ILC1-Like Cells Are a Potent *In Vitro* Precursor of KIR^+^ NK Cells

Another exciting discovery we made, was while working with CB ILC1-like cells. A comparison of the transcriptome of CB ILC1-like cells to CD56^bright^ and CD56^dim^ NK cells, both formerly located within group 1 ILCs (8), revealed that most differentially expressed genes within CB ILC1-like cells compared to NK cells were T cell-associated genes, such as *CD5* and *CCR7* (21). Of note, this was previously reported in tonsillar ILC1 (33) and PB ILC1 (32, 34). As most CB ILC1-like cells were immature, we suspected ILC1-like cells to be precursors. Indeed, within bulk and single cell cultures CB ILC1-like cells differentiated into fully functional mature NKG2A^-^KIR^+^NK cells exhibiting a broad KIR repertoire, which was not seen in parallel cultured CD56^bright^ NK cells (21). Hence, CB ILC1-like cells represent a novel NK precursor (NKP) able to generate NK cells with complex KIR repertoires *in vitro*.

Previously reported NK cell precursors have been described to express CD34 or CD117 (35), however CB ILC1-like cells lack both (21). Our data suggests that NK development is not based on a linear (35) but rather a branched model (36, 37) and in fact, CB ILC1-like NKPs and circulating CD56^bright^ NK cells might both contribute to the CD56^dim^ NK cell pool. In this model, CD56^bright^ NK cells would maintain NKG2A:CD94 expression and up-regulate KIR, while CB ILC1-like NKPs downregulate NKG2A, but keep KIR expression (21). We further observed a significant decline in CB ILC1-like frequencies and cell count with increased gestational age (21), which is in line with ‘ILC-poiesis’ suggesting circulating ILCs to migrate into tissues (22). Taking the findings together, we propose that fetal ILC1-like cells enter the bloodstream from hematopoietic sites before migrating into secondary lymphoid tissues *via* CCR7. Following maturation into NK cells in the SLNs, they would be release into circulation post-birth to contribute to the formation of complex KIR repertoires.

#### 2.1.5 Future Directions on Human ILCs

CB ILCs intrigue and challenge me, as the data we generated was unexpected, but totally fascinating at the same time: CB ILCs are dominantly immature, have a different transcriptional identity and harbor a very potent NK precursor. Many times, we had to think out-side the box. Our research raised several questions: Where do circulating ILCs come from and what is their purpose? Where do they go and how? When and how does the transcriptome of human ILCs change – directly post-birth or during ageing? Furthermore, more insight into the developmental relationship between CB ILC1-like cells and CD56^dim^ NK cells is needed. And I can honestly say that I would be delighted to help to address these questions in the future.

## 3 Challenges

### 3.1 Studying as a Mature Student

During my undergraduate degree, I was deeply worried about my chances to graduate at all. Even though evidence suggests that more mature students do well in academia due to their unique attributes (38), I could not see my advantages during the first two years. Furthermore, I knew studies also suggest students admitted *via* waiting list quotas were less likely to do well in medical studies (39), which is understandable when they have to worry about finances or childcare (38). I must admit, I have never looked for mentoring programs or support groups during my undergraduate degree or graduate studies. However, it might be nice to have tutors for mature students, who also entered as mature students. I had no idea about life as a student and I spend the first year figuring out how to study, as I was out of school for 6 years. I felt pressured, I simply forgot a lot of things, which my fellow students - coming right from school - still remembered. Therefore, I felt the need to study very hard to succeed. When I later talked to some of my fellow students, I realized they were also lost at first. For some, it was the first time living alone and away from home, and the study curriculum was also hard for them.

It took me a long time to realize that my “maturity” should not be considered a disadvantage, but rather as an opportunity. When I applied to a two-month internship in Oxford the PI chose me since I already had lab experience and he specifically asked if I could adjust quickly to the new lab. I had an amazing time. Given my technical background, I work very efficiently, paralleling experiments, and adapting cost-effectiveness. I also had the impression during my interview with Oxford that my unusual CV was seen as a strength rather than a weakness. Oxford is also a very international place with very interesting people, who all have their unique stories, so my CV was just one of many. I also had a tutor from my college, who met with me regularly to discuss how I am doing and discuss my future plans. During my PhD, I was more open about my feelings. Interestingly, I had the same issues as other (most of the time younger) PhD students, as PhD work can feel like a constant up and down between success, failure, and pressure.

### 3.2 What About Children?

Since turning 30, I am constantly asked when I will get married and have children. On the one hand, one feels rather pressured to give in to the society’s needs of starting a family, but as a female researcher, I have doubts. Especially when people tell you – as they told me – that I would never become a successful scientist and might not be able to start my own group, because I am not young enough and most importantly because I am female. I know that this burning question ‘when is the best time to start a family?’ is not only in my head, but in many heads of women all over the world across all ranges of age.

Unfortunately, the fear is not completely unjustified. Evidence exists mothers, but not fathers, are penalized during job recruitments (40). To make matters worse, mothers who spend 3-month in parental leave to re-start their career early are discriminated with significantly lower invited interviews compared to mothers with 12-month parental leave, while men`s parental leave duration had no impact (41) suggesting women are at a disadvantage anyway. Especially in academia where contracts are mostly short-term, and pressure is very high to accomplish many things in short time periods. On the bright side, international awareness is growing and topics such as the question on how mothers are treated in academia is now a matter of public debate and was recently also featured in *Nature* (42). It is also encouraging that some grant givers have turned to a policy that enables grant extension for scientists based on the number of children. Nonetheless, if one feels a passion for science this should not discourage anybody to pursue a scientific career. In fact, my advice would be to turn it around and take hurdles as additional motivation just like I did, proving to yourself that you can do it.

## 4 Conclusion

After studying for seven years (2013–2021), I have successfully completed my PhD at the age of 33 in May 2021. My advice would be to speak up and to actively look for people, who might be able to help or ‘just’ listen. I recommend being yourself and acting upon your fundamental principles. I learned that you are judged by others whatever you do, but you should not define yourself based on this. Since I have my PhD, I feel as if the glass ceiling has been broken and I feel empowered. However, there are still times, when I felt and still feel pressured to make up for the time I worked as a technical assistant. Nevertheless, I am a very passionate, enthusiastic scientist and who knows what the future may hold, but third parties should not set your limits. I studied very hard for being where I am today, and I think it was worth the effort. I hope by sharing my story, I can encourage others to believe in themselves.

## Author Contributions

The author confirms being the sole contributor of this work and has approved it for publication.

## Funding

Article fee was funded by the Open-Access-Fonds of the Heinrich-Heine Universität.

## Conflict of Interest

The author declares that the research was conducted in the absence of any commercial or financial relationships that could be construed as a potential conflict of interest.

## Publisher’s Note

All claims expressed in this article are solely those of the authors and do not necessarily represent those of their affiliated organizations, or those of the publisher, the editors and the reviewers. Any product that may be evaluated in this article, or claim that may be made by its manufacturer, is not guaranteed or endorsed by the publisher.

## References

[1] Disteldorf EM Krebs CF Paust H-J Turner J-E Nouailles G Tittel A. CXCL5 Drives Neutrophil Recruitment in TH17-Mediated Gn. J Am Soc Nephrol (2015) 26(1):55–66. doi: 10.1681/ASN.2013101061 24904089 PMC4279732

[2] Krebs CF Kapffer S Paust H-J Schmidt T Bennstein SB Peters A. MicroRNA-155 Drives TH17 Immune Response and Tissue Injury in Experimental Crescentic Gn. J Am Soc Nephrol (2013) 24(12):1955–65. doi: 10.1681/ASN.2013020130 PMC3839549 23949802

[3] Paust H-J Riedel J-H Krebs CF Turner J-E Brix SR Krohn S. CXCR3+ Regulatory T Cells Control TH1 Responses in Crescentic Gn. J Am Soc Nephrol (2016) 27(7):1933–42. doi: 10.1681/ASN.2015020203 PMC4926966 26534920

[4] Schmidt T Paust H-J Krebs CF Turner J-E Kaffke A Bennstein SB. Function of the Th17/Interleukin-17a Immune Response in Murine Lupus Nephritis. Arthritis Rheumatol (2015) 67(2):475–87. doi: 10.1002/art.38955 25385550

[5] Turner J-E Krebs C Tittel AP Paust H-J Meyer-Schwesinger C Bennstein SB. IL-17a Production by Renal γδ T Cells Promotes Kidney Injury in Crescentic Gn. J Am Soc Nephrol (2012) 23(9):1486–95. doi: 10.1681/ASN.2012010040 PMC3431412 22797181

[6] Turner J-E Paust H-J Bennstein SB Bramke P Krebs C Steinmetz OM. Protective Role for CCR5 in Murine Lupus Nephritis. Am J Physiol - Renal Physiol (2012) 302(11):F1503–15. doi: 10.1152/ajprenal.00382.2011 22442210

[7] Bennstein SB. Unraveling Natural Killer T-Cells Development. Front Immunol (2018) 8(1950). doi: 10.3389/fimmu.2017.01950 PMC5767218 29375573

[8] Spits H Artis D Colonna M Diefenbach A Di Santo JP Eberl G. Innate Lymphoid Cells -a Proposal for Uniform Nomenclature. Nat Rev Immunol (2013) 13(2):145–9. doi: 10.1038/nri3365 23348417

[9] Vivier E Artis D Colonna M Diefenbach A Di Santo JP Eberl G. Innate Lymphoid Cells: 10 Years on. Cell (2018) 174(5):1054–66. doi: 10.1016/j.cell.2018.07.017 30142344

[10] Bernink JH Peters CP Munneke M te Velde AA Meijer SL Weijer K. Human Type 1 Innate Lymphoid Cells Accumulate in Inflamed Mucosal Tissues. Nat Immunol (2013) 14(3):221–9. doi: 10.1038/ni.2534 23334791

[11] Mjösberg JM Trifari S Crellin NK Peters CP van Drunen CM Piet B. Human IL-25- and IL-33-Responsive Type 2 Innate Lymphoid Cells Are Defined by Expression of CRTH2 and CD161. Nat Immunol (2011) 12:1055. doi: 10.1038/ni.2104 21909091

[12] Cella M Fuchs A Vermi W Facchetti F Otero K Lennerz JKM. A Human NK Cell Subset Provides an Innate Source of IL-22 for Mucosal Immunity. Nature (2009) 457(7230):722–5. doi: 10.1038/nature07537 PMC3772687 18978771

[13] Hoorweg K Peters CP Cornelissen F Aparicio-Domingo P Papazian N Kazemier G. Functional Differences Between Human NKp44(–) and NKp44(+) RORC(+) Innate Lymphoid Cells. Front Immunol (2012) 3:72. doi: 10.3389/fimmu.2012.00072 22566953 PMC3342004

[14] Bennstein SB Uhrberg M. Biology and Therapeutic Potential of Human Innate Lymphoid Cells. FEBS J (2021). doi: 10.1111/febs.15866 33837637

[15] Lim AI Li Y Lopez-Lastra S Stadhouders R Paul F Casrouge A. Systemic Human ILC Precursors Provide a Substrate for Tissue ILC Differentiation. Cell (2017) 168(6):1086–100.e10. doi: 10.1016/j.cell.2017.02.021 28283063

[16] Simoni Y Fehlings M Kløverpris HN McGovern N Koo S-L Loh CY. Human Innate Lymphoid Cell Subsets Possess Tissue-Type Based Heterogeneity in Phenotype and Frequency. Immunity (2017) 46(1):148–61. doi: 10.1016/j.immuni.2016.11.005 PMC7612935 27986455

[17] Hazenberg MD Haverkate NJE van Lier YF Spits H Krabbendam L Bemelman WA. Human Ectoenzyme-Expressing ILC3: Immunosuppressive Innate Cells That Are Depleted in Graft-Versus-Host Disease. Blood Adv (2019) 3(22):3650–60. doi: 10.1182/bloodadvances.2019000176 PMC6880892 31751473

[18] Nagasawa M Germar K Blom B Spits H. Human CD5+ Innate Lymphoid Cells Are Functionally Immature and Their Development From CD34+ Progenitor Cells Is Regulated by Id2. Front Immunol (2017) 8(1047). doi: 10.3389/fimmu.2017.01047 PMC5583608 28912776

[19] Forsberg A Bengtsson M Eringfält A Ernerudh J Mjösberg J Jenmalm MC. GATA Binding Protein 3+ Group 2 Innate Lymphoid Cells are Present in Cord Blood and in Higher Proportions in Male Than in Female Neonates. J Allergy Clin Immunol (2014) 134(1):228–30.e2. doi: 10.1016/j.jaci.2014.01.027 24636083

[20] Vely F Barlogis V Vallentin B Neven B Piperoglou C Ebbo M. Evidence of Innate Lymphoid Cell Redundancy in Humans. Nat Immunol (2016) 17(11):1291–9. doi: 10.1038/ni.3553 PMC5074366 27618553

[21] Bennstein SB Weinhold S Manser AR Scherenschlich N Noll A Raba K. Umbilical Cord Blood-Derived ILC1-Like Cells Constitute a Novel Precursor for Mature KIR+NKG2A- NK Cells. eLife (2020) 9:e55232. doi: 10.1101/2020.01.24.918318 32657756 PMC7358013

[22] Lim AI Di Santo JP. ILC-Poiesis: Ensuring Tissue ILC Differentiation at the Right Place and Time. Eur J Immunol (2019) 49(1):11–8. doi: 10.1002/eji.201747294 30350853

[23] Bianca Bennstein S Riccarda Manser A Weinhold S Scherenschlich N Uhrberg M. OMIP-055: Characterization of Human Innate Lymphoid Cells From Neonatal and Peripheral Blood. Cytometry Part A (2019) p. 427–30. doi: 10.1002/cyto.a.23741 30887685

[24] Bennstein SB Scherenschlich N Weinhold S Manser AR Noll A Raba K. Transcriptional and Functional Characterization of Neonatal Circulating ILCs. Stem Cells Trans Med (2021) 10(6):867–82. doi: 10.1002/sctm.20-0300 PMC8133339 33475258

[25] Cella M Otero K Colonna M. Expansion of Human NK-22 Cells With IL-7, IL-2, and IL-1β Reveals Intrinsic Functional Plasticity. Proc Natl Acad Sci (2010) 107(24):10961–6. doi: 10.1073/pnas.1005641107 PMC2890739 20534450

[26] Lass A Weiser W Munafo A Loumaye E. Leukemia Inhibitory Factor in Human Reproduction. Fertil Steril (2001) 76(6):1091–6. doi: 10.1016/S0015-0282(01)02878-3 11730732

[27] Kojima K Kanzaki H Iwai M Hatayama H Fujimoto M Narukawa S. Expression of Leukaemia Inhibitory Factor (LIF) Receptor in Human Placenta: A Possible Role for LIF in the Growth and Differentiation of Trophoblasts. Hum Reprod (1995) 10(7):1907–11. doi: 10.1093/oxfordjournals.humrep.a136205 8583009

[28] Oliveira-Nascimento L Massari P Wetzler LM. The Role of TLR2 in Infection and Immunity. Front Immunol (2012) 3:79. doi: 10.3389/fimmu.2012.00079 PMC3342043 22566960

[29] Robbins JR Zeldovich VB Poukchanski A Boothroyd JC Bakardjiev AI. Tissue Barriers of the Human Placenta to Infection With Toxoplasma Gondii. Infect Immun (2012) 80(1):418–28. doi: 10.1128/IAI.05899-11 PMC3255695 22083708

[30] Yokota Y Mansouri A Mori S Sugawara S Adachi S Nishikawa S-I. Development of Peripheral Lymphoid Organs and Natural Killer Cells Depends on the Helix–Loop–Helix Inhibitor Id2. Nature (1999) 397(6721):702–6. doi: 10.1038/17812 10067894

[31] Xu W Cherrier DE Chea S Vosshenrich C Serafini N Petit M. An Id2(RFP)-Reporter Mouse Redefines Innate Lymphoid Cell Precursor Potentials. Immunity (2019) 50(4):1054–68.e3. doi: 10.1016/j.immuni.2019.02.022 30926235 PMC6477155

[32] Li S Morita H Sokolowska M Tan G Boonpiyathad T Opitz L. Gene Expression Signatures of Circulating Human Type 1, 2 and 3 Innate Lymphoid Cells. J Allergy Clin Immunol (2019) 143(6):2321–5. doi: 10.1016/j.jaci.2019.01.047 30825467

[33] Bjorklund AK Forkel M Picelli S Konya V Theorell J Friberg D. The Heterogeneity of Human CD127+ Innate Lymphoid Cells Revealed by Single-Cell RNA Sequencing. Nat Immunol (2016) 17(4):451–60. doi: 10.1038/ni.3368 26878113

[34] Roan F Stoklasek TA Whalen E Molitor JA Bluestone JA Buckner JH. CD4+ Group 1 Innate Lymphoid Cells Form a Functionally Distinct ILC Subset That is Increased in Systemic Sclerosis. J Immunol (Baltimore Md 1950) (2016) 196(5):2051–62. doi: 10.4049/jimmunol.1501491 PMC4761490 26826243

[35] Freud AG Keller KA Scoville SD Mundy-Bosse BL Cheng S Youssef Y. NKp80 Defines a Critical Step During Human Natural Killer Cell Development. Cell Rep (2016) 16(2):379–91. doi: 10.1016/j.celrep.2016.05.095 PMC4970225 27373165

[36] Cichocki F Grzywacz B Miller JS. Human NK Cell Development: One Road or Many? Front Immunol (2019) 10(2078). doi: 10.3389/fimmu.2019.02078 PMC6727427 31555287

[37] Michel T Poli A Cuapio A Briquemont B Iserentant G Ollert M. Human CD56 Bright NK Cells: An Update. J Immunol (2016) 196(7):2923–31. doi: 10.4049/jimmunol.1502570 26994304

[38] Santos L Bago J Baptista A Ambrósio S Fonseca H Quintas H. Academic Success of Mature Students in Higher Education: A Portuguese Case Study. Eur J Res Educ Learn Adults (2016) 7:57–73. doi: 10.3384/rela.2000-7426.rela9079

[39] Meyer H Zimmermann S Hissbach J Klusmann D Hampe W. Selection and Academic Success of Medical Students in Hamburg, Germany. BMC Med Educ (2019) 19(1):23. doi: 10.1186/s12909-018-1443-4 30651098 PMC6335698

[40] Hipp L. Do Hiring Practices Penalize Women and Benefit Men for Having Children? Experimental Evidence From Germany. Eur Sociol Rev (2019) 36(2):250–64. doi: 10.1093/esr/jcz056

[41] Hipp L. Damned If You do, Damned If You Don’t? Experimental Evidence on Hiring Discrimination Against Parents With Differing Lengths of Family Leave. SocArXiv (2018). doi: 10.31235/osf.io/qsm4x

[42] Powell K. How Mothers Get Penalized in the Scientific Race. Nature (2021) 595:611–3. doi: 10.1038/d41586-021-01993-x

